# Intrathecal delivery of frataxin mRNA encapsulated in lipid nanoparticles to dorsal root ganglia as a potential therapeutic for Friedreich’s ataxia

**DOI:** 10.1038/srep20019

**Published:** 2016-02-17

**Authors:** Joseph F. Nabhan, Kristy M. Wood, Varada P. Rao, Jeffrey Morin, Surya Bhamidipaty, Timothy P. LaBranche, Renea L. Gooch, Fazli Bozal, Christine E. Bulawa, Braydon C. Guild

**Affiliations:** 1 Rare Disease Research Unit, Pfizer, 610 Main Street, Cambridge, MA 02139, USA; 2 Global Biotherapeutics, Pfizer, 610 Main Street, Cambridge, MA 02139, USA; 3 Comparative Medicine, Pfizer, 1 Burtt Rd, Andover, MA 01810, USA; 4 Drug Safety Research and Development, Pfizer, 1 Burtt Rd, Andover, MA 01810, USA

## Abstract

In Friedreich’s ataxia (FRDA) patients, diminished frataxin (FXN) in sensory neurons is thought to yield the predominant pathology associated with disease. In this study, we demonstrate successful usage of RNA transcript therapy (RTT) as an exogenous human FXN supplementation strategy *in vitro* and *in vivo*, specifically to dorsal root ganglia (DRG). Initially, 293 T cells were transfected with codon optimized human *FXN* mRNA, which was translated to yield FXN protein. Importantly, FXN was rapidly processed into the mature functional form of FXN (mFXN). Next, *FXN* mRNA, in the form of lipid nanoparticles (LNPs), was administered intravenously in adult mice. Examination of liver homogenates demonstrated efficient FXN LNP uptake in hepatocytes and revealed that the mitochondrial maturation machinery had efficiently processed all FXN protein to mFXN in ~24 h *in vivo*. Remarkably, greater than 50% mFXN protein derived from LNPs was detected seven days after intravenous administration of FXN LNPs, suggesting that the half-life of mFXN *in vivo* exceeds one week. Moreover, when FXN LNPs were delivered by intrathecal administration, we detected recombinant human FXN protein in DRG. These observations provide the first demonstration that RTT can be used for the delivery of therapeutic mRNA to DRG.

Friedreich’s ataxia (FRDA) is an autosomal recessive disease caused by an intronic trinucleotide (GAA) expansion in intron 1 of the *FXN* (frataxin) gene^1^. FRDA is predominantly a neurodegenerative disease^2^, but pathology also manifests in multiple tissues including the heart and pancreas^3^,^4^. Expansions in the *FXN* gene have been shown to cause epigenetic changes^5^ and formation of R-loops^6^ that hinder the transcriptional machinery, ultimately yielding diminished levels of *FXN* transcript and protein. In FRDA patients, FXN protein levels have been shown to be reduced in all tested cell types^7^. Although *FXN* knockout is lethal^8^, diminished levels yield pathology only in specific cell types, including select neurons, cardiomyocytes and pancreatic islets, for reasons that are not understood. In the central nervous system (CNS), progressive degeneration leads to disease, therefore therapies that access the CNS are highly desirable.

The function of FXN is still a subject of debate but the protein’s primary role is activation of iron-sulfur (Fe-S) cluster biogenesis in the mitochondrial matrix^9^. Data have also been reported supporting a role for FXN as an iron chaperone^10^. Regardless of its precise function, it has been established that in FRDA patients, levels of FXN in peripheral tissues drop to ~5–30% of those in non-carrier healthy individuals^11^. In affected individuals, such a decrease in cellular concentration of FXN yields pathology. Interestingly, heterozygous patients have ~50% FXN compared to non-carriers and do not show any pathology. There is no disease-modifying therapy for FRDA so treatment options remain limited, but increasing FXN levels to those in carriers of the pathogenic *FXN* intronic expansion is desirable and could be therapeutic. A number of small molecules, such as histone deacetylase inhibitors^12^ and nicotinamide^13^, and large molecules, such as engineered transcription activator-like effectors^14^,^15^, were reported as FXN upregulation approaches for FRDA therapy. FXN protein supplementation^16^ and viral gene transfer^17^,^18^ are other potential strategies for therapy that are currently being explored.

RNA transcript therapy (RTT) is an mRNA replacement/supplementation approach through encapsulation of exogenous mRNA molecules in nanoparticles. RTT is currently being pursued for multiple therapeutic applications^19^ and has led to major investments in biopharmaceutical companies^20^. In proof-of-principle example studies, RTT was shown to successfully rescue a lethal genetic knockout mouse model^21^ and to be useful for delivery of an mRNA encoding a therapeutic protein in a model of disease^22^, suggesting that it can be utilized as a replacement approach in inherited recessive diseases such as FRDA. In this study, we explored RTT by intravenous and intrathecal lipid nanoparticle (LNP) based delivery of recombinant human *FXN* (h*FXN)* mRNA. We present evidence that h*FXN* mRNA can be efficiently translated *in vitro* upon cellular transfection and *in vivo* when administered systemically, and that the corresponding protein is processed into the mature, functional form (mFXN). Importantly, we find that the maturation machinery is not limiting, and the *in vivo* half-life of mFXN protein is long, in excess of one week. We further tested intrathecal delivery and uptake of h*FXN* mRNA in dorsal root ganglia (DRG), a disease-affected and primary site of pathology in FRDA^2^,^23^. These results demonstrate the potential utility of LNP-based delivery of h*FXN* mRNA as a supplementation therapy to treat FRDA and for other diseases of the central nervous system where DRG are implicated in pathology.

## Results

### *In vitro* transcribed mRNA encoding *FXN* is efficiently translated into protein that is processed to yield functional mature FXN

**Figure 1:**
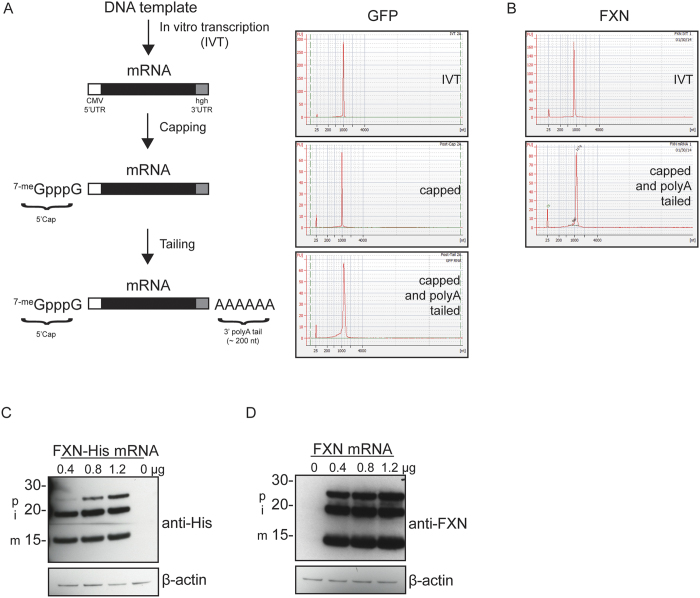
*FXN* mRNA is translated and processed to yield mature functional FXN in transfected cells. (**A**) Schematic of the process used for mRNA generation. Linearized DNA template was used to generate the primary IVT product containing a cytomegalovirus (CMV) 5′ untranslated region (UTR) and a human growth hormone (hgh) 3′ UTR, and then subjected to 5′ capping and 3′ polyA tailing reactions to yield the final transcript. Bioanalyzer traces of IVT, capped product, and final polyA-tailed transcript are shown for (**A**) *GFP* and (**B**) *FXN* mRNA. (**C**) 293 T cells were transfected with the indicated amounts of FXN-6xHis or (**D**) FXN mRNA for 24 h before lysis and immunoblotting with the indicated antibodies. Anti-β-actin immunoblotting was used as a lane-loading control. p, i, and m indicate precursor, intermediate, and mature FXN protein immunopositive signals, respectively.

Plasmid DNA encoding GFP (Green Fluorescent Protein) containing a 5′ T7 RNA polymerase promoter was linearized and used to generate mRNA enzymatically with an *in vitro* transcription (IVT) system. GFP sequence was flanked with a 5′ untranslated region (5′ UTR) from the cytomegalovirus (CMV) promoter and a 3′ human growth hormone (hgh) UTR. This reaction was followed by enzymatic capping and polyA tailing reactions to add a 5′Cap-1 structure and a 3′ polyA tail, respectively (Fig. 1A). mRNA purity and size were determined using a Bioanalyzer at each step of the process. PolyA tails were estimated to be ~200 nucleotides (nt) long after the tailing reactions. To ascertain that *in vitro* transcribed GFP mRNA could be translated into GFP protein, we transfected 293 T cells with increasing amounts of mRNA using two transfection reagents, Fugene 6 and Lipofectamine 2000 (Lipo2000; Fig. S1A). Cells transfected using Fugene 6 did not yield GFP fluorescence; however, a robust signal was observed with Lipo2000 24 h post-transfection. Cells were then harvested and examined for GFP protein signal by immunoblotting (Fig. S1B). An anti-GFP immunopositive signal was detected in cells transfected with GFP mRNA using Lipo2000 that was dose-dependent with the amount of transfected mRNA. We then generated *FXN* mRNA by IVT using a linearized plasmid containing codon-optimized human FXN (Fig. S2A), which encodes a protein sequence identical to consensus (Fig. S2B). This was followed by the same capping and polyA tailing reactions detailed in Fig. 1A. h*FXN* mRNA was analyzed following IVT and capping/polyA tailing (Fig. 1B). We asked if *FXN* mRNA could be translated in transfected cells, and if the corresponding protein could be processed to yield the mature functional form that resides in the mitochondrial matrix^24^,^25^. HexaHis-tagged h*FXN* mRNA, which was also generated and analyzed as above (data not shown), was used to transfect 293 T cells for 24 h. Three anti-His immunopositive bands were detected corresponding to the approximate sizes of precursor FXN (FXN 1–210; 23.1 KDa), intermediate FXN (FXN 42–210; 19 KDa) and mature FXN (FXN 81–210; 14.2 KDa), indicating that exogenously administered *FXN* mRNA is translated and processed in the mitochondria (Fig. 1C). Similarly, FXN protein derived from mRNA encoding untagged FXN and transfected into 293 T cells was translated and processed into intermediate (iFXN) and mature FXN protein (Fig. 1D). Note that FXN protein derived from transfected mRNA was in much excess of endogenous protein, which could not be detected at the exposure time utilized in Fig. 1D, but was still processed into i- and mFXN. These data suggest that the mitochondrial maturation machinery is not rate limiting.

### Generation of LNPs encapsulating *FXN* mRNA and *in vivo* administration

**Figure 2:**
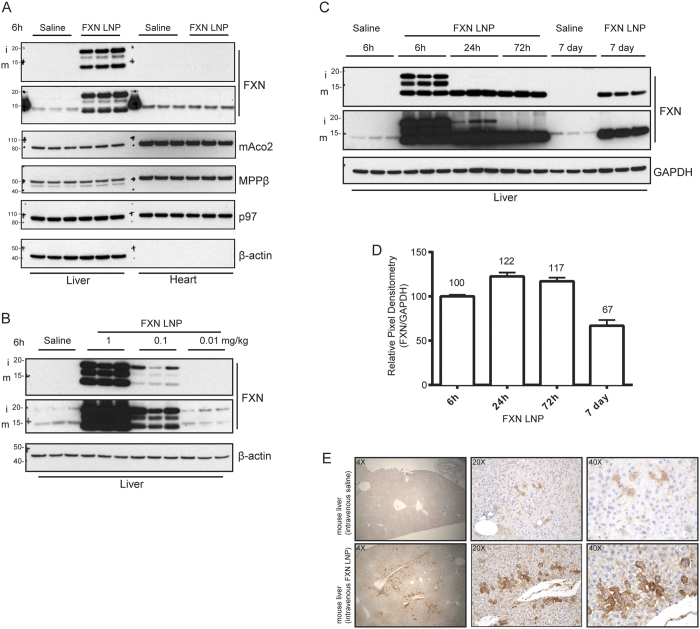
*FXN* mRNA delivered using LNP vehicle *in vivo* is efficiently translated and processed in liver. (**A**) Mice were injected IV with FXN LNPs or saline solution as control (3 animals per group). Tissues, including liver and heart, were collected 6 h post-injection and frozen before preparation of homogenates for western analysis. Immunoblotting was carried out with the indicated antibodies. Two antibodies targeting FXN were used to confirm the immunopositive signal corresponding to FXN (upper panel: abcam ab110328; lower panel: Proteintech 14147-01-AP). p97 and β-actin were used as lane-loading controls. (**B**) Various concentrations of FXN LNP were tested by IV administration. Lysates derived from livers 6 h after IV injection of LNP or saline solution (3 animals per group) were prepared and analyzed by immunoblotting with the indicated antibodies. Two antibodies targeting FXN were used to confirm the immunopositive signal corresponding to FXN (upper panel: abcam ab110328; lower panel: Proteintech 14147-01-AP). (**C**) Mice were injected IV with FXN LNP or saline solution and animals were necropsied at different timepoints post-LNP administration. Liver homogenates were prepared and analyzed by western blotting with the indicated antibodies. Two antibodies targeting FXN were used to confirm the immunopositive signal corresponding to FXN (upper panel: abcam ab110328; lower panel: Proteintech 14147-01-AP). (**D**) Pixel densitometry analysis of the FXN LNP –derived mFXN immunopositive signals at 6 h, 24 h, 72 h, and 7 days post-administration. Densitometries were normalized relative to levels at 6 h. (**E**) Anti-FXN IHC staining of mouse livers 24 h after administration of FXN LNPs or saline solution. Three magnifications (4×, 20×, and 40×) of the same region are shown.

LNPs were formulated by microfluidic mixing of an ethanolic lipid solution (molar ratio 55:10:32.5:2.5 of MC3:DSPC:Chol:PEG) and mRNA in citrate buffer, pH 4 (Fig. S3A). Lipids were combined with mRNA at a total flow rate of 20 mL/min to yield a 30:1 final weight ratio of lipid:mRNA in 25% ethanol. Nanoparticles were dialyzed into PBS, pH 7.4 and concentrated by Amicon centrifugal filters where needed. LNPs encapsulating h*FXN* mRNA measured ~75–85 nm in diameter on average with greater than 95% mRNA encapsulation (Fig. S3B). To test translation of h*FXN* mRNA *in vivo*, we first carried out tail vein injections of FXN LNPs in CD-1 mice at 0.1 mg/kg *FXN* mRNA. Biodistribution studies of intravenously-administered LNPs had previously shown predominant uptake in liver tissue^26^. We analyzed liver lysates, harvested 6 h post –LNP administration, by immunoblotting for FXN with different anti-FXN commercial antibodies to examine translation of exogenously administered h*FXN* mRNA (Fig. 2A). We also included heart lysates in our analysis because heart is an FRDA disease-affected tissue. In saline-treated mice, a single faint band corresponding to ~15 KDa was detected. In contrast, three bands were detected in our analysis of liver lysates from animals that received h*FXN* mRNA. Two bands corresponded to the predicted sizes of human iFXN and mFXN indicating successful import and maturation of FXN in the mitochondria. Importantly, levels of mature human FXN protein greatly exceeded that of endogenous mouse mFXN, which displayed slower electrophoretic mobility compared to the immunopositive band of human mFXN. At 6 h post–IV (intravenous) administration of FXN LNPs, an ~16 KDa band was also detected, which may represent a second intermediate that precedes final maturation of the protein. Precursor FXN was not observed, likely due to the rapid processing of FXN into the intermediate as was previously reported^27^. These data indicated that h*FXN* mRNA is efficiently translated and that the FXN maturation machinery can process excess FXN protein. We also confirmed that the principal processing enzyme MPPβ is expressed in liver and heart tissues. To ascertain that increased FXN levels in liver do not adversely influence other mitochondrial proteins, we examined mitochondrial aconitase (Aco2) by immunoblotting. No changes were observed in the levels of Aco2 in lysates containing excess FXN compared to controls. No signal corresponding to exogenous FXN was observed in heart lysates, indicating the LNP IV administration is unlikely to deliver h*FXN* mRNA to cardiac tissue under the current context. In these experiments we used antibodies against β-actin and p97/VCP as lane loading controls. As expected in heart lysates, β-actin is not expressed therefore no signal was detected.

To determine if the observed partial processing of human FXN to the mature form was due to overloading of the mitochondrial maturation machinery, we tested IV administration of decreasing dose levels of *FXN* mRNA LNPs (1.00, 0.10, and 0.01 mg/kg). Analysis of liver lysates from mice that received 1 mg/kg and 0.1 mg/kg h*FXN* mRNA yielded a strong immunopositive signal indicative of efficient translation of exogenously administered mRNA (Fig. 2B). However, even at the lowest level of administered LNPs (0.01 mg/kg), a residual amount of iFXN was still observed suggesting incomplete processing. We then explored FXN protein levels over an extended time period after IV administration of FXN LNPs at the 1 mg/kg dose level. 24 h after FXN LNP injection, a predominant majority of exogenous FXN protein in liver was processed to mature form, and by 72 h all of the detected FXN was in the form of mature FXN (Fig. 2C). The absence of intermediate FXN signal at 72 h suggests that all of the exogenously administered h*FXN* mRNA was translated and processed. Remarkably, when we examined levels of FXN 7 days post –LNP administration, FXN levels were in great excess of endogenous FXN protein. This is consistent with the previously reported long half-life of mature FXN *in vitro*^27^. To estimate human FXN half-life *in vivo* in mice administered FXN LNPs, we quantified the human mFXN immunopositive signal from liver lysates. Mature FXN levels increased at 24 h due to maturation of translated h*FXN*. 7 days after FXN LNP administration, >60% of the human mFXN signal remained in liver lysates. We further tested heart lysates 6 h and 24 h post LNP IV injection and compared liver levels of FXN in LNP recipient animals to those in crude human brain lysates (Fig. S4A). No human FXN protein was detected in mouse heart lysates but liver FXN levels, normalized to either lane-loading controls GAPDH or p97/VCP, were in obvious excess of endogenous human brain FXN levels. We then compared LNP-derived FXN levels in mouse liver lysates to human tissues implicated in FRDA pathology, including cerebellum and heart (Fig. S4B). Human FXN levels were much higher in liver lysates from mice administered FXN LNPs compared to normal human tissue levels. Importantly, in liver lysates from IV administered FXN LNP mice, levels of NFS1 and ISCU2, both FXN interaction partners required for Fe-S biosynthesis^28^,^29^, were unaltered despite the very robust increase in FXN levels (Fig. S4B). Altogether, the data suggest that conceptually it would be possible to increase FXN levels using FXN LNPs beyond endogenous levels without an obvious deleterious effect.

We next examined FXN signal by immunohistochemistry (IHC) in liver tissue 24 h after IV injection of FXN LNPs (Fig. 2E), using an antibody validated to detect endogenous FXN (Fig. S5). A clear increase in FXN signal was observed in corresponding paraffin-embedded liver sections compared to those from controls. As expected, at 40× magnification, the signal was in the form of puncta in the cytoplasm, consistent with mitochondrial localization of FXN protein. Importantly, and as shown by immunoblotting (Fig. 2C), the intensity of the FXN immunopositive signal in animals given IV FXN LNPs was higher than that observed for endogenous FXN protein in control tissues (Fig. 2E). However, IHC did not inform on maturation of LNP-derived human FXN protein, since epitopes targeted by the anti-FXN antibody reside in the mFXN region, which is shared with i- and pFXN. We therefore generally favored immunoblotting over IHC analysis.

### Intrathecal administration of firefly luciferase and *FXN* mRNA encapsulated in LNPs

**Figure 3:**
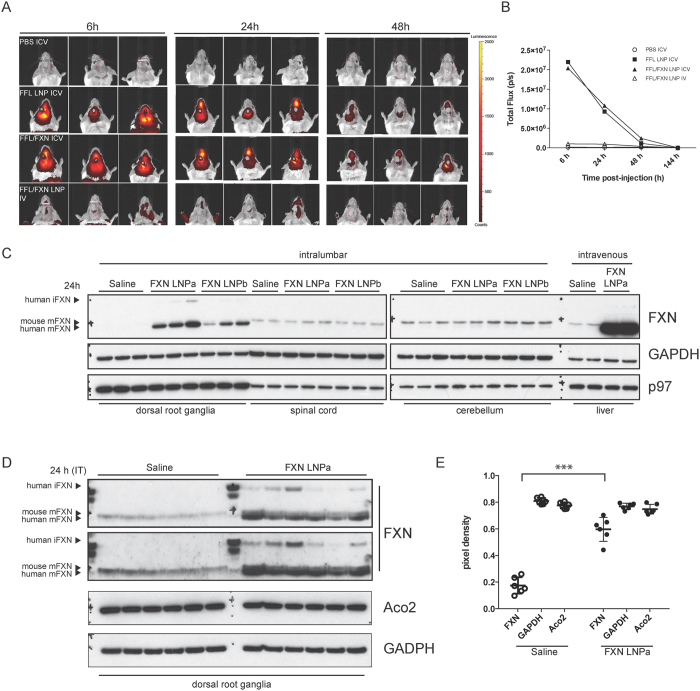
Uptake of firefly luciferase and frataxin mRNA LNPs in the CNS. (**A**) Cannulated mice were administered saline solution or the indicated LNP formulation ICV or IV, as noted. Animals were injected D-luciferin intraperitoneally at 6 h, 24 h, or 48 h post-LNP administration and imaged using IVIS. Representative bioluminescence images are shown at each time point. (**B**) Bioluminescence expressed as the average of flux (photon/sec; p/s) in 3 animals per time point per group is shown plotted against time (h). (**C**) DRG, spinal cord, and cerebella from mice injected with saline solution, FXN LNPa or LNPb (3 animals per group) intrathecally were harvested 24 h post-administration. Additionally, 2 animals were injected IV with saline solution or FXN LNPa and livers were harvested. Tissue homogenates were prepared and analyzed by immunoblotting with the indicated antibodies. Arrowheads indicate the positions of immunopositive bands corresponding to human iFXN, mouse mFXN, and human mFXN. (**D**) Mice were intrathecally injected with h*FXN* LNPa or saline solution (6 animals per group), and DRG were harvested 24 h post-administration. DRG lysates were analyzed by immunoblotting with the indicated antibodies. To enhance detection of the endogenous mouse FXN signal in DRG lysates, anti-FXN (Proteintech 14147-01-AP) was diluted at 1:500. Two exposures of the anti-FXN immunoblot are shown. Arrowheads indicate the positions of immunopositive bands corresponding to human iFXN, mouse mFXN, and human mFXN. (**E**) anti-FXN immunopositive signals corresponding to mouse mFXN in saline solution –injected controls and human mFXN in FXN LNPa injected animals were quantified by pixel densitometry. Aco2 and GAPDH immunopositive signals were also quantified. Pixel densities for all three proteins from each animal group are shown (open circles for saline injected animals; full circles for FXN LNPa injected animals) with corresponding standard deviations. A two-tailed Student’s *t*-test was carried out to compare densitometries for each protein between the two groups. Significant differences between the two groups are indicated; ***P < 0.0001.

To test LNP uptake in the CNS, we administered LNPs that encapsulated only firefly luciferase (FFL) mRNA (0.2 mg/kg), a co-formulation of *FFL* mRNA and h*FXN* mRNA (1:1 ratio; 0.2 mg/kg), or PBS (pH 7.4) control into the left lateral ventricle via cannula surgically installed in BALB/c mice. FFL and FXN were co-delivered in LNPs to investigate if h*FXN* mRNA encapsulation may influence LNP uptake in the CNS. Mice were then injected with D-luciferin and imaged on an IVIS Lumina at the indicated time points (Fig. 3A). Animals that received FFL LNP and FFL/FXN LNP formulations displayed a strong bioluminescence signal at 6 h and 24 h. Consistent with the short half-life of FFL protein, the luminescence signal was significantly diminished by 48 h post-administration (Fig. 3B). As a positive control for the formulations, FFL/FXN LNPs were also administered by tail-vein administration and livers were imaged to confirm luciferase signal. As expected, a strong signal was detected in the liver for the IV administered group (data not shown) and no luminescence was detected in the brain for these animals. Importantly, no differences in signal intensity and distribution were observed between animals administered FFL alone or the FFL/FXN co-formulation suggesting that the nucleic acid sequence corresponding to h*FXN* mRNA did not influence LNP uptake in the CNS.

In FRDA, DRG are sensitive to diminished levels of FXN and represent a major site of pathology^23^,^30^,^31^. Having established uptake of LNPs in the CNS, we asked if FXN LNP particles could be delivered to DRG to increase FXN levels. In order to administer LNPs to a region proximal to DRG, we injected FXN LNP formulations intrathecally into the lower lumbar region (L4, L5) of the mouse spine. 24 h after injection of FXN LNPs, DRG, spinal cord, and cerebella were harvested and analyzed (Fig. 3C, intralumbar). We also investigated if increasing PEG content (4%) and thus reducing LNP size (60 nm diameter; LNPb) increased cellular uptake, compared to our standard formulation of 2.5% PEG content (85 nm diameter; LNPa). In saline injected controls, mouse FXN levels in DRG at steady state were hard to detect by western blotting, suggesting that they are very low. Animals that received LNPa and LNPb showed a robust increase in mature FXN derived from exogenously administered and translated human h*FXN* mRNA. Formulations containing the larger size particles with lower PEG content (LNPa) yielded more FXN signal than LNPb. No LNP-derived human FXN protein was detected in spinal cord and cerebellar lysates. As positive controls, a group of animals were injected with LNPa formulation IV and livers were harvested for analysis. As expected, a large increase in FXN was observed in the livers of corresponding animals (Fig. 3C, intravenous). In all instances where FXN LNPs were administered, exogenous FXN protein was almost completely processed to the mature functional form, indicating that the mitochondrial maturation machinery is capable of handling the additional FXN load. In an attempt to quantify the amount of LNP-derived human mFXN relative to endogenous mouse mFXN, we repeated the above experiment with a larger number of animals (6 mice per group) using the LNPa formulation. 24 h after administration of FXN LNP or saline solution as control, DRG were harvested and analyzed by western blotting. We increased the concentration of anti-FXN antibody (Proteintech 14147-01-AP) to 1:500 and extended exposure of membranes to film in order to detect endogenous mouse mFXN in saline solution -administered DRG lysates. This led to an increase in background noise but permitted detection of endogenous mouse mFXN in DRG. LNP-derived FXN was in excess of endogenous mouse FXN, and LNP administration had no effect on the Fe-S requiring protein Aco2 (Fig. 3D). We then quantified levels of human mFXN in the FXN LNPa group compared to endogenous mouse mFXN in the saline group. On average, LNP-derived human mFXN levels were ~3-fold higher than mouse mFXN in the control group (Fig. 3E). Levels of Aco2 and GAPDH in DRG lysates were not significantly different between the saline and FXN LNP groups.

## Discussion

FRDA is an inherited progressive neurodegenerative disease presenting initially with ataxia in childhood, followed by loss of ambulation, dysarthria, and hypertrophic cardiomyopathy. The root cause of pathology is a reduction in levels of FXN. Generally, three different therapeutic strategies have been pursued: 1) upregulation of endogenous FXN levels, 2) anti-oxidant therapy to alleviate oxidative stress, a secondary consequence of low levels of FXN, and 3) FXN replacement via gene therapy or protein supplementation. We tested a novel supplementation strategy, RTT, that utilizes nanoparticles as vehicles to deliver h*FXN* mRNA. Importantly, when cultured cells were transfected with h*FXN* mRNA, the recombinant protein product was efficiently generated and processed by the mitochondrial processing machinery to yield iFXN and mFXN. We set out to investigate if *in vivo* IV administration of FXN LNPs to mice can lead to translation of the encapsulated mRNA. Examination of liver tissue lysates showed translation of h*FXN* mRNA at 6 h but limited processing within that period. When we examined liver tissue lysates at 24 h or later, almost all of the translated *FXN* mRNA was processed into the mature form. LNP-derived FXN, despite being in much excess of endogenous mouse FXN, was fully processed and mFXN protein was long-lived with a half-life of ~1 week. We further administered FXN LNPs intrathecally and detected robust mRNA translation and FXN protein processing in DRG – the amount of FXN protein derived from LNPs was in significant excess of endogenous levels. Collectively, these data support the feasibility of FXN RTT as a potential therapy for FRDA, particularly since data shown here suggest that the mitochondria can tolerate and process excess levels of FXN protein.

Several FXN supplementation strategies have been previously described. A fusion protein composed of the transactivator of transcription (TAT) protein transduction domain, which is thought to facilitate cell penetration, and FXN was tested in mice. TAT-FXN was shown to process properly *in vitro* and to rescue the aconitase functional deficit associated with *FXN* knockout (KO) in fibroblasts, and to increase survival in *FXN* cardiac KO mice^16^. Concerns though remain about TAT peptide toxicity and immunogenicity^32^, and efficiency of endosomal escape of internalized TAT fusion proteins^33^ to permit import into mitochondria. More recently, multiple publications described AAV (adeno-associated virus) – based delivery of FXN as a gene therapy approach. Systemic injection of cardiotropic AAVrh10 viruses containing the human *FXN* gene in conditional heart and skeletal muscle *FXN* knockout mice (MCK-Cre) led to robust expression of FXN and rescue of associated phenotypes, as well as increased survival^18^. Another group utilized intraperitoneal injection of AAV9 encoding human *FXN* in MCK-Cre mice and showed increased survival and improvement in cardiac function compared to controls^17^.

This report demonstrates, for the first time, delivery of exogenous human FXN to DRG *in vivo*. As detailed in this report, h*FXN* mRNA encapsulated in LNPs were injected intrathecally and successfully yielded a robust increase in mFXN levels. Previous reports have primarily focused on delivery of exogenous FXN to cardiac tissue. In this study, IV injection of FXN LNPs did not lead to increased cardiac levels of FXN, however delivery and translation of mRNA to the myocardium using LNPs have been previously shown^22^. Intrathecal delivery of FXN LNPs produced a pronounced signal of FXN in DRG although no augmentation over endogenous levels was observed in spinal cord or cerebellum. The DRG are supplied by fenestrated capillaries and lack a tight blood-nerve barrier^34^; this may render them more accessible to the current nanoparticle formulation. Alternatively, perhaps the current formulation delivers minimal quantities of nanoparticles to spinal and cerebellar neurons yielding low levels of human FXN that are masked by the endogenous murine FXN. It is likely that delivery of mRNA nanoparticles to some tissues is greatly impeded by non-fenestrated and non-permeable vascular endothelia, as had been previously suggested^35^. Whereas delivery of mRNA nanoparticles has been shown to hard-to-access tissues such as the heart through intramyocardial injection of the formulation^22^, IV administration is unlikely to permit delivery to the heart or other FRDA disease-affected tissues^36^.

Further testing is needed to confirm that recombinant FXN is functional in DRG. Although FRDA mouse models have been generated, their CNS pathology is absent or mild when GAA repeats are introduced into the *FXN* locus or severe where *FXN* is knocked out^37^, making efficacy studies difficult. One previously reported *FXN* KO model utilized NSE-Cre to generate a conditional KO in neurons, however animals die shortly post-partum^38^, which does not permit intrathecal delivery of FXN LNPs to assess efficacy. It would be interesting to test FXN RTT in an FRDA mouse model that presents CNS pathology in adulthood, such as the YG8R mouse^39^, albeit the associated disease phenotype is modest. A model that manifests more severe CNS pathology in adulthood and within a shorter timeframe, such as an inducible knockdown, would be highly desirable to test FXN supplementation approaches for functional rescue. If FXN LNPs are efficacious by intrathecal administration, it is possible that injections would only be required once a month or less often, given the slow decay in mFXN and the ability of cells to tolerate an excess amount of FXN without yielding a deleterious effect. Although such an approach would be invasive, given the requirement for repeat administration, intrathecal catheters connected to surgically implanted reservoirs that can be refilled with a therapeutic agent are currently being developed^40^, which may minimize the risks associated with administration. Altogether, this study provides a method that enables recombinant mRNA delivery in the form of nanoparticles to DRG for disease treatment, such as in the case of FRDA and spinal muscular atrophy^41^, or for other applications.

## Materials and Methods

### mRNA preparation, purification and cell transfection

A codon-optimized DNA encoding full-length human precursor FXN, FXN-6xHis (C-terminally tagged HexaHis), or GFP were subcloned into a bacterial plasmid 3′ of a T7 promoter (DNA2.0). The coding sequence was flanked by a 5′ sequence from the untranslated region (5′ UTR) from the immediate early region of the cytomegalovirus and a 3′ sequence from the human growth hormone (3′ UTR). *In vitro* transcription (IVT) followed by capping and polyA tailing of IVT products were carried out using the mScript system (Cellscript) following the manufacturer’s recommendations. All reactions were done at 37 °C and typical reaction times were: 30 min for IVT, 30 min for capping, and 60 min for polyA tailing. IVT reaction and final products were purified using the RNeasy MinElute Cleanup Kit (Qiagen). Concentrations were determined by Abs260 measurements (Nanodrop) and purity was examined using the Bioanalyzer (Agilent 2100). For mRNA transfections, 293 T cells were seeded at 200,000 cells per well in a 12-well plate and transfected with the indicated amounts of GFP, FXN, or FXN-6xHis mRNA and 4 μl Lipofectamine 2000 (Life Technologies). 24 h after transfection, cells were washed briefly with PBS and lysed in 100 μl lysis buffer (0.5% Igepal CA-630 from Sigma, 50 mM TrisCl pH 7.5, 150 mM NaCl) supplemented with protease and phosphatase inhibitor cocktails (Roche).

### Formulation of LNPs and mRNA encapsulation

Lipid stock solutions of (6 Z, 9 Z, 28 Z, 31 Z)-heptatriaconta-6,9,28,31-tetraen-19-yl 4-(dimethylamino)butanoate (DLin-MC3-DMA), 1,2-distearoyl-sn-glycero-3-phosphocholine (DSPC; Avanti), cholesterol (Chol; Avanti), and 1,2-Dimyristoyl-sn-glycerol methoxypolyethylene glycol (DMG-PEG2k; NOF) were prepared in ethanol at a concentration of 50 mM. For formulation, lipids were combined to yield a molar ratio of 55:10:32.5:2.5 (DLin-MC3-DMA:DSPC:Chol:DMG-PEG2k) at a total lipid concentration of 25 mM. mRNA was prepared in 10 mM sodium citrate, pH 4 at a concentration that yielded a final weight ratio of 30:1 of lipid:mRNA. mRNA and lipids were combined by microfluidic mixing (Precision Nanosystems, Nanoassemblr) at a 20 mL/min flow rate and a 3:1 volumetric ratio of mRNA:lipid. Formulations were immediately diluted in PBS to an ethanol concentration of 12.5% and then dialyzed (Slide-a-lyzer, Thermofisher; 20 kD cutoff) overnight in PBS, pH 7.4. Where needed, formulations were concentrated with centrifugal filters (Amicon, 100 kD cutoff). LNPs were filtered with a 0.22 μm syringe filter and stored at 2–8 °C until use. Particle size and polydispersity index (PDI) was characterized by dynamic light scattering (Malvern Instruments Zetasizer) with formulations typically yielding a z-average size of 75–85 nm and a PDI < 0.2. Formulation concentration and encapsulation was determined by a modified Ribogreen assay (Life Technologies) where standards and samples were prepared with and without 1% Triton X-100. Encapsulation was determined by measuring the fluorescence in samples with Triton X-100 (total mRNA) and samples without Triton X-100 (free mRNA). % Encapsulation ((total mRNA – free mRNA) / total mRNA) was typically found to be greater than 95%.

### *In vivo* administration of LNPs

Eight-week old female BALB/c mice (Jackson Laboratory) were received with intra-cerebroventricular (ICV) cannulae surgically implanted by the vendor. Cannulae were stereotaxically placed with the following coordinates: Medial-lateral (left) 1 mm, rostral-caudal −0.4 mm. The cannula is a 23 gauge stainless steel tube with an internal diameter of 0.24 mm and an external diameter of 0.46 mm, with the injection cannula extending 2.2 mm below a plastic pedestal. ICV injections were carried out under isoflurane anesthesia using sterile technique. 5 μL of LNP formulation (0.2 mg/kg) or sterile phosphate buffered saline (PBS) was injected into the left lateral ventricle at a rate of 1 μL/minute. Animals were allowed to recover in their cage following administration and were monitored daily. As controls a cohort of mice was injected intravenously with the firefly luciferase-LNP formulation (0.2 mg/kg) at a volume of 100 μL. Mice were housed in individually ventilated cages with *ad libitum* water and diet. All procedures on mice were carried out following guidelines and protocols reviewed and approved by Pfizer Institutional Animal Care and Use Committee (IACUC). For IT/intralumbar injections, experiments were carried out at Biomere (Worcester, MA USA). Briefly, seven-week old BALB/c female mice were anesthetized with isoflurane inhalation (1–4%, to effect). Animals were then shaved and the injection site (L4-L5 or L5-L6) was palpated. A reflexive flick of the tail indicated puncture of the dura and confirmed intrathecal placement ahead of LNP (or sterile saline solution) administration. Animals were housed in clear polycarbonate cages with contact bedding, inside a procedure room, and fed Mouse Diet 5058 and spring water acidified with 1 N HCl to a targeted pH of 2.5–3 *ad libitum*.

### *In vivo* bioluminescence imaging

Six hours after the administration of the LNP formulations, mice were injected intraperitoneally (i.p.) with a 150 mg/kg solution of luciferin (XenoLight D-Luciferin, Perkin Elmer). Cranial region was imaged on the IVIS Lumina XR (Perkin Elmer, Hopkinton, MA) 15 minutes after i.p. injection (Field of View B, Auto exposure, F stop = 1, Open filter). Imaging was performed under isoflurane anesthesia using a heated stage. Liver region was also imaged using the IVIS Lumina XR (Field of View C, Auto exposure, F stop = 1, Open filter). Animals were imaged additionally at 24 and 48 hours after LNP administration. Luminescent intensity in regions of interest drawn around the brain and liver were quantified using Living Image analysis software (Perkin Elmer).

### Tissue harvesting and immunoblot analysis

Mice were euthanized by CO_2_ inhalation and tissues were collected, immediately snap frozen in liquid nitrogen and stored at −80 °C before homogenization. To homogenize tissues and extract protein, stainless steel beads (Qiagen) and lysis buffer (0.5% Igepal630, 50 mM Tris pH 7.5, 150 mM NaCl, Roche mini-tablet protease inhibitor cocktail) were applied to frozen tissues placed in Eppendorf Safe-Lock microtubes and subjected to two rounds of bead beating (26/sec; 2 min per round) in the Qiagen TissueLyser II. A small sample of vortexed homogenate was further diluted in lysis buffer, centrifuged for 10 min at 21,000 × g at 4 C, and subjected to a protein 660 assay (Thermofisher). Final lysates were resolved (5–10 μg/lane) on 4–12% Nupage BisTris gels (Life Technologies) using MES buffer, before transfer to nitrocellulose membranes on the Transblot Turbo system (Biorad). Membranes were then subjected to western blotting using the indicated antibodies. Antibodies used in this study and corresponding dilutions are listed in Supplementary Table 1.

### Immunohistochemistry

IHC on formalin-fixed paraffin embedded mouse tissues was performed on an automated Immunostainer (Bond RX, Leica Biosystems), using the Bond Polymer Refine Detection kit (Leica Biosystems) as instructed by the manufacturer. 4 μm liver sections were tested via IHC. Briefly, following de-paraffinization and rehydration, antigen retrieval was performed on the Leica Bond Rx immunostainer using Heat Induced Epitope Retrieval 1 (citrate buffer, pH 6). The slides were placed in this solution and heated to 60 °C for 20 minutes. Afterwards, the slides were cooled and gently washed in Bond wash solution. Slides were then incubated with 3% hydrogen peroxide for 5 minutes to quench endogenous peroxidase activity and subsequently rinsed with Bond wash solution before incubation in primary anti-FXN antibody (1:150; abcam ab110328) for 60 minutes. A post-primary IgG linker reagent was then applied to localize mouse antibodies, and the substrate chromogen, 3,3′-Diaminobenzidine tetrahydrochloride hydrate (DAB) allowed for visualization of the complex via a brown precipitate. Finally, hematoxylin (blue) counterstaining allowed the visualization of cell nuclei. A slide subjected to the same treatment but lacking primary antibody was used to control for any background staining due to the retrieval and detection steps. FXN protein immunostaining was assessed qualitatively based on chromogenic staining intensity.

## Additional Information

**How to cite this article**: Nabhan, J. F. *et al*. Intrathecal delivery of frataxin mRNA encapsulated in lipid nanoparticles to dorsal root ganglia as a potential therapeutic for Friedreich's ataxia. *Sci. Rep.* **6**, 20019; doi: 10.1038/srep20019 (2016).

## Supplementary Material

Supplementary Information
