# Supplementary material for: Intrathecal delivery of frataxin mRNA encapsulated in lipid nanoparticles to dorsal root ganglia as a potential therapeutic for Friedreich’s ataxia
Source: Sci Rep. 2016 Feb 17;6:20019. doi: 10.1038/srep20019 (PMC4756688; doi:10.1038/srep20019)
Supplement: Supplementary Information [file srep20019-s1.pdf]

Intrathecal delivery of frataxin mRNA encapsulated in lipid nanoparticles to dorsal root ganglia as a potential therapeutic for Friedreich's ataxia

Joseph F Nabhan[1*], Kristy M Wood[2], Varada P Rao[3], Jeffrey Morin[3], Surya Bhamidipaty[4], Timothy P LaBranche[4], Renea L Gooch[1], Fazli Bozal[1], Christine E Bulawa[1], Braydon C Guild[2]

[1] Pfizer Biotherapeutics, Rare Disease Research Unit, 610 Main Street, Cambridge, MA

[2] Pfizer Global Biotherapeutics, 610 Main Street, Cambridge, MA

[3] Pfizer Comparative Medicine, 1 Burtt Rd, Andover, MA

[4] Pfizer Drug Safety Research and Development, 1 Burtt Rd, Andover, MA

* Corresponding author: joseph.nabhan@pfizer.com, +1(617)674-7101

SUPPLEMENTARY INFORMATION

Supplementary Figures

Figure S1. GFP mRNA transfected into 293T cells are translated to yield functional protein. A) 293T cells seeded in 12 well plates were transfected with increasing amounts (0.2, 0.4, and 0.8 μg) of GFP mRNA using Fugene 6 (Promega) or Lipofectamine 2000 (Lipo2000; Life Technologies). Cells were imaged ~24 h after transfection using an EVOS microscope equipped with an EVOS GFP Light Cube (Thermofisher). Fugene 6 transfections did not yield any detectable fluorescence even at the highest amount of transfected mRNA (0.8 μg GFP mRNA transfection shown). Transfections with Lipo2000 yielded an increasing amount of fluorescence indicative of successful transfection and translation of GFP mRNA. Representative fluorescence images are shown with corresponding bright field captures at 10x magnification. B) Cells transfected as detailed above were harvested 24 h post-transfection and lysates were analyzed by western blotting with the indicated antibodies.

Figure S2. Optimized FXN cDNA sequence used as a template for FXN IVT. A) The FXN coding sequence (630 nucleotides) was codon optimized to improve translation efficiency. The sequence of the optimized sequence is shown (top) with the corresponding translated amino acid sequence (bottom). B) NCBI's blastp tool (http://blast.ncbi.nlm.nih.gov/Blast.cgi) was used to align the consensus sequence of FXN (uniprot ID Q16595 / NCBI RefSeq NP_000135.2; Query) with the translation of codon-optimized FXN (Sbjct).

Figure S3. LNP composition and formulation process. A) Lipid molar percentages and weight ratio used in LNPs and overview of the LNP formulation process. B) Example trace of final particle size and distribution by intensity for LNPs.

Figure S4. Comparison of LNP-derived human FXN levels with human brain tissue levels. A) Lysates derived from liver (1 animal) and heart (3 animals) homogenates of mice administered IV saline or FXN LNPs were analyzed by immunoblotting with the indicated antibodies. Also included in the analysis is an equal amount of total human brain lysate (abcam ab29466). GAPDH and p97/VCP antibodies were used as lane loading controls. Multiple exposures are shown for the anti-FXN immunoblots. B) Liver lysates from animals injected with saline solution or FXN LNPs and human tissue lysates (cerebellar vermis, right cerebellum, left cerebellum, cerebral meninges, and heart; Genetex) were analyzed by immunoblotting with the indicated antibodies.

Figure S5. Anti-FXN IHC staining of mouse brain, heart, and liver tissue sections. Immunostaining was carried out as detailed in the Methods using 1:250 anti-FXN (abcam ab110328) followed by secondary antibody (upper panel) or with secondary antibody alone (lower panel). Representative images are shown. Magnified insets are shown for upper panel images.

Supplementary Tables

Supplementary Table 1. List of antibodies and corresponding dilutions used for western blotting.

Figure S1

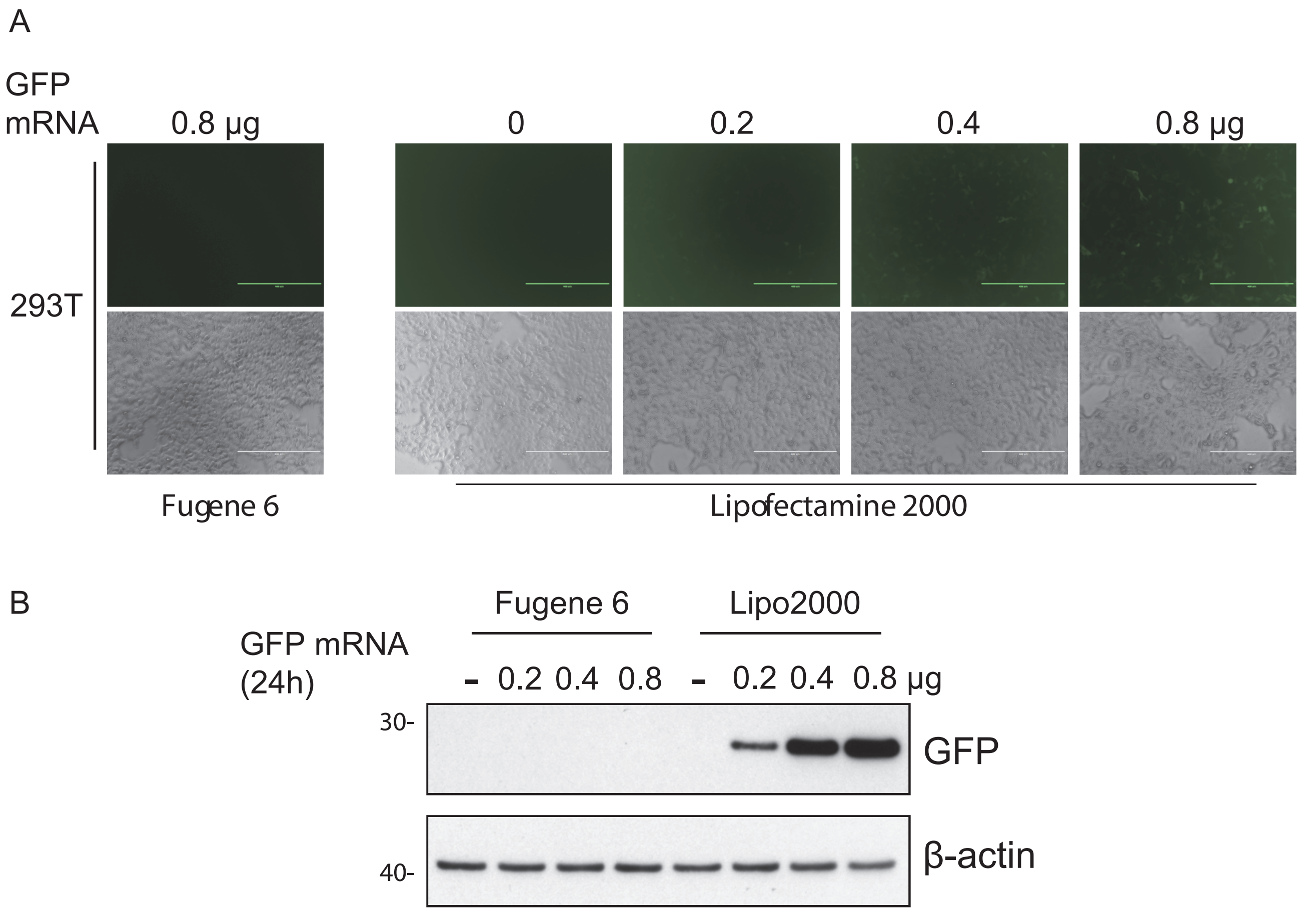

A
GFP mRNA
0.8 µg
0
0.2
0.4
0.8 µg
293T
Fugene 6
Lipofectamine 2000
B
Fugene 6
Lipo2000
GFP mRNA (24h)
- 0.2 0.4 0.8 - 0.2 0.4 0.8 µg
30-
GFP
40-
β-actin

Figure S2

A

Codon-optimized human FXN sequence (630 nt)

ATGTGGACCCTGGGACGACGCGCCGTGGCGGGACTGCTGGCTTCACCGAGCCCGGCTCA
GGCACAAACTCTGACTCGCGTTCCAAGGCCTGCCGAACTCGCCCCGCTTTGCGGACGGA
GAGGCTTGAGAACGGACATTGACGCCACTTGTACTCCAAGGCGCGCCAGCTCGAATCAGC
GCGGACTGAATCAGATCTGGAACGTGAAGAAGCAATCCGTCTACCTCATGAACCTCCGGA
AGTCGGGAACGCTCGGCCACCCGGGCAGCCTTGATGAAACTACCTACGAACGGCTGGCG
GAGGAAACTCTGGATTCCCTGGCCGAATTCTTCGAGGATCTGGCGGACAAACCATACACC
TTTGAGGACTACGATGTCTCCTTCGGGTCCGGTGTGTTGACCGTGAAGCTGGGGGGCGAC
CTGGGTACCTACGTCATCAACAAACAGACCCCTAACAAGCAAATCTGGCTCTCGTCCCCGT
CATCAGGACCCAAGCGGTATGACTGGACCGGGAAGAATTGGGTGTACAGCCATGACGGA
GTGTCGCTTCATGAGTTGCTGGCCGCGGAGCTCACTAAAGCACTCAAGACCAAACTCGAC
CTGTCATCGCTGGCATACTCTGGAAAGGATGCTTAG

Translation of codon-optimized human FXN (210 AA)

MWTLGRRAVAGLLASPSPAQAQTLTRVPRPAELAPLCGRRGLRTDIDATCTPRRASSNQRGL
NQIWNVKKQSVYLMNLRKSGTLGHPGSLDETTYERLAEETLDSLAEFFEDLADKPYTFEDYDV
SFGSGVLTVKLGGDLGTYVINKQTPNKQIWLSSPSSGPKRYDWTGKNWVYSHDGVSLHELLA
AELTKALKTKLDLSSLAYSGKDA

B

Alignment of translation of codon-optimized FXN (Query) with consensus FXN amino acid sequence uniprot ID Q16595 / NCBI RefSeq NP_000135.2 (Sbjct)

frataxin, mitochondrial isoform 1 preproprotein [Homo sapiens]
Sequence ID: ref|NP_000135.2| Length: 210 Number of Matches: 1
▶ See 7 more title(s)

Range 1: 1 to 210 GenPept Graphics ▼ Next Match ▲ Previous Match

Score	Expect	Method	Identities	Positives	Gaps
431 bits(1108)	2e-151	Compositional matrix adjust.	210/210(100%)	210/210(100%)	0/210(0%)

```
Query  1    MWTLGRRAVAGLLASPSPAQAQTLTRVPRPAELAPLCGRRGLRTDIDATCTPRRASSNQR  60
            MWTLGRRAVAGLLASPSPAQAQTLTRVPRPAELAPLCGRRGLRTDIDATCTPRRASSNQR
Sbjct  1    MWTLGRRAVAGLLASPSPAQAQTLTRVPRPAELAPLCGRRGLRTDIDATCTPRRASSNQR  60

Query  61   GLNQIWNVKKQSVYLMNLRKSGTLGHPGSLDETTYERLAEETLDSLAEFFEDLADKPYTF  120
            GLNQIWNVKKQSVYLMNLRKSGTLGHPGSLDETTYERLAEETLDSLAEFFEDLADKPYTF
Sbjct  61   GLNQIWNVKKQSVYLMNLRKSGTLGHPGSLDETTYERLAEETLDSLAEFFEDLADKPYTF  120

Query  121  EDYDVSFGSGVLTVKLGGDLGTYVINKQTPNKQIWLSSPSSGPKRYDWTGKNWVYSHDGV  180
            EDYDVSFGSGVLTVKLGGDLGTYVINKQTPNKQIWLSSPSSGPKRYDWTGKNWVYSHDGV
Sbjct  121  EDYDVSFGSGVLTVKLGGDLGTYVINKQTPNKQIWLSSPSSGPKRYDWTGKNWVYSHDGV  180

Query  181  SLHELLAAELTKALKTKLDLSSLAYSGKDA  210
            SLHELLAAELTKALKTKLDLSSLAYSGKDA
Sbjct  181  SLHELLAAELTKALKTKLDLSSLAYSGKDA  210
```

Figure S3

A

LNP Composition

Lipid	(mol%)
DLin-MC3-DMA (MC3)	55.0
DSPC	10.0
Cholesterol (Chol)	32.5
PEG_{2k} -DMG (PEG)	2.5
wt ratio of total lipid:mRNA	30:1

LNP Formulation Process

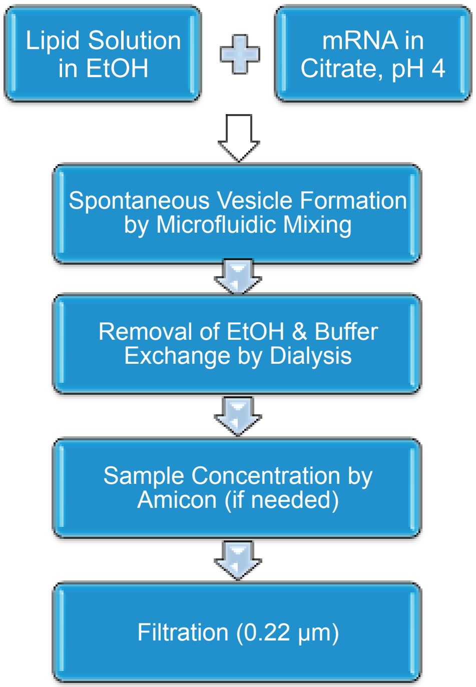

B

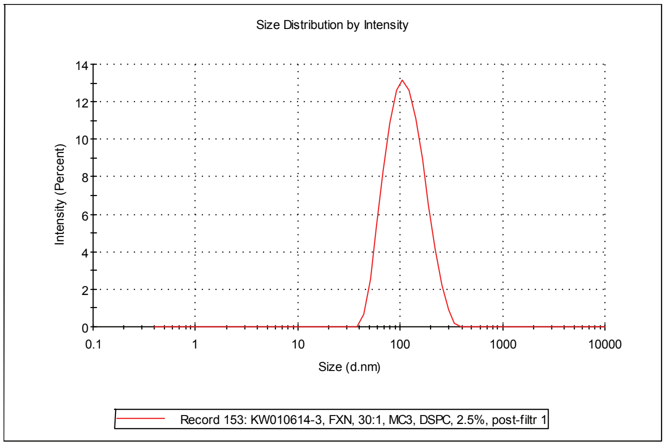

Figure S4

A

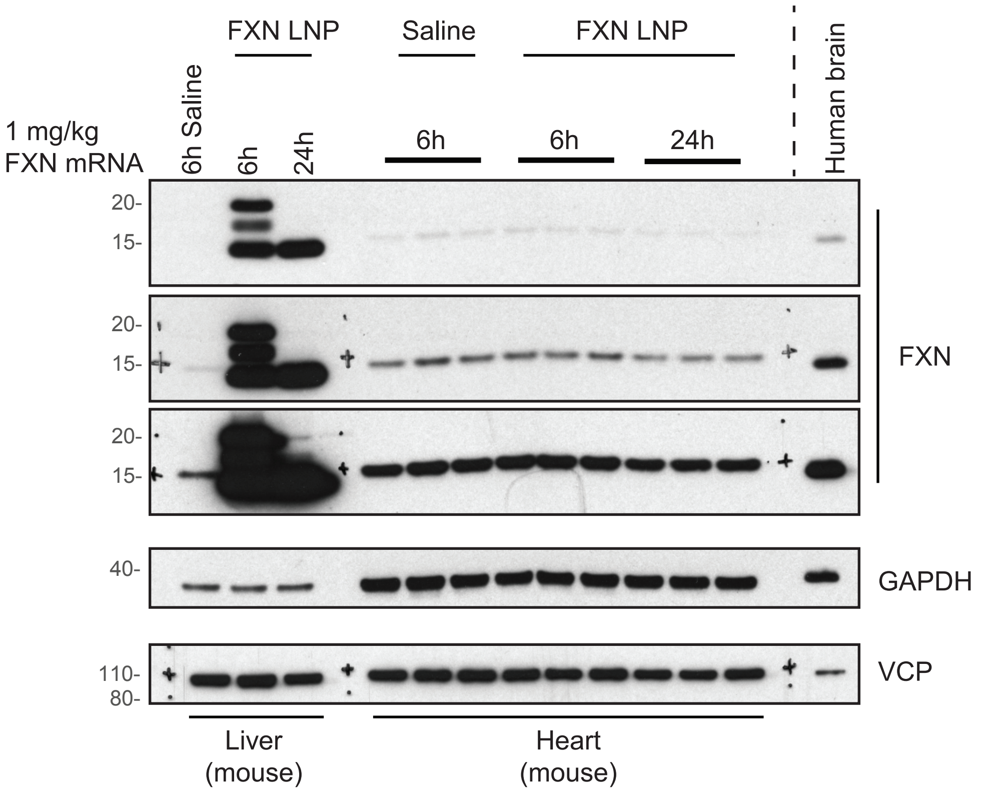
FXN LNP
Saline
FXN LNP
6h Saline
1 mg/kg
FXN mRNA
6h
24h
6h
6h
24h
Human brain
20-
15-
20-
15-
20-
15-
FXN
40-
GAPDH
110-
80-
VCP
Liver
(mouse)
Heart
(mouse)

B

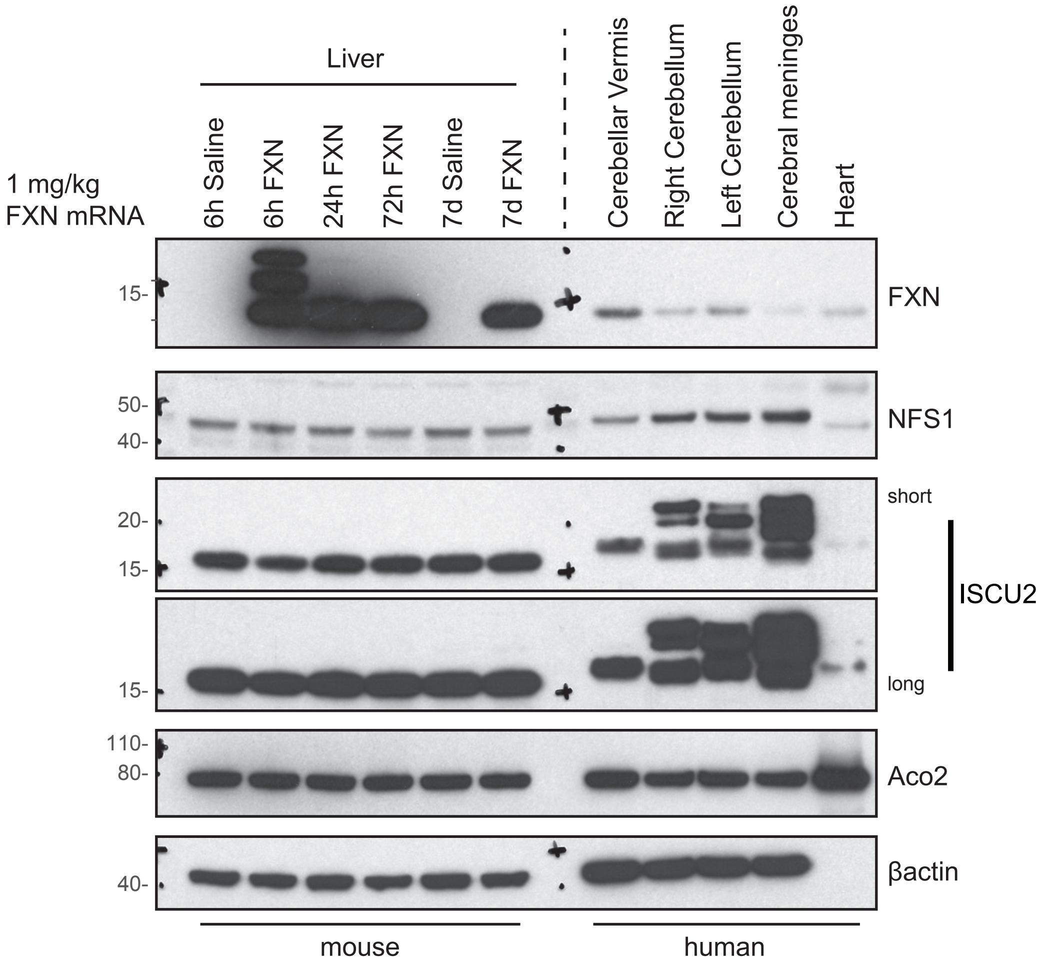
Liver
1 mg/kg
FXN mRNA
6h Saline
6h FXN
24h FXN
72h FXN
7d Saline
7d FXN
Cerebellar Vermis
Right Cerebellum
Left Cerebellum
Cerebral meninges
Heart
15-
FXN
50-
40-
NFS1
short
20-
15-
ISCU2
15-
long
110-
80-
Aco2
40-
βactin
mouse
human

Figure S5

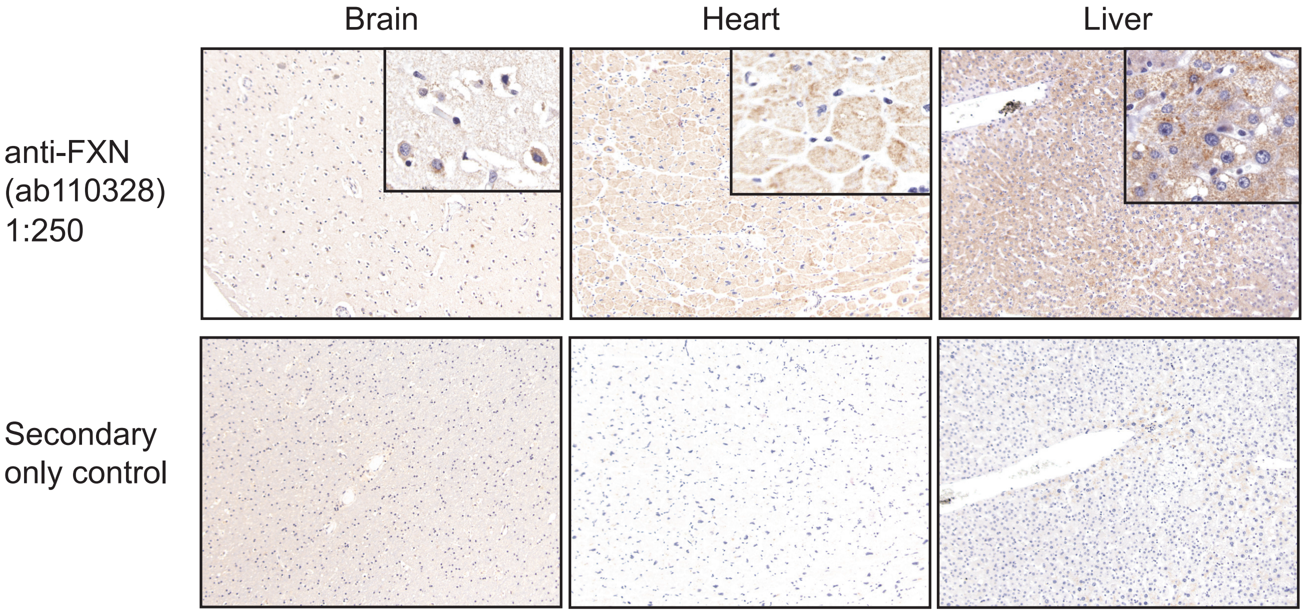
Brain
Heart
Liver
anti-FXN
(ab110328)
1:250
Secondary
only control

Supplementary Table 1. List of antibodies and corresponding dilutions used for western blotting.

Protein name	Observed MW (KDa)	Source	Cat #	Ab dilution
Aco2	~80	abcam	ab129069	1:6000
FXN	14-21	abcam	ab110328	1:1000
FXN	14-21	Proteintech	14147-01-AP	1:1000; 1:500 (Fig. 3D)
GAPDH	38	Cell Signaling	8884S	1:12,000
GFP	~28	Cell Signaling	2956S	1:1000
ISCU2	15-20	Proteintech	14812-1-AP	1:6000
MPPβ	~50	Proteintech	16064-1-AP	1:2000
NFS1	~50	Proteintech	15370-1-AP	1:2000
p97/VCP	~100	Cell Signaling	2649S	1:1000
β-actin	42	Cell Signaling	5125S	1:12,000
HexaHis	n/a	Amersham	27-4710-01	1:1000
